# Fellowships, Grants, & Awards

**Published:** 2004-12

## NIGMS National Centers for Systems Biology

The National Institute of General Medical Sciences (NIGMS) currently supports the analysis of complex biological systems through investigator-initiated research project grants. The resources needed to conduct the multifaceted, multidisciplinary projects that may be required to achieve significant advances in these complex areas may be beyond the scope of the typical R01 or P01 grant. Therefore, this request for applications (RFA) presents an opportunity for applicants to assemble large teams of investigators from diverse disciplines that may not be possible with other funding mechanisms.

The biomedical sciences have undergone a fundamental shift in the conceptual and technical approaches that can be applied to certain problems of profound importance. These problems center on understanding the behavior of biological systems whose function is the product of spatial and temporal ordering of myriad interacting components. Modeling approaches are being used to understand the orderly development of biological pattern in organisms such as *Drosophila* and *Caenorhabditis elegans*, and at the clinical level, new approaches are being explored to understand the integrated activity of tissues and organs.

Part of the impetus for systems-scale approaches rests on advances in acquiring data of the necessary quality and quantity to permit computational modeling. Among the most striking examples are the availability of complete DNA sequences for hundreds of organisms, including humans, and the availability of high-throughput instrumentation for analyses of gene function such as gene expression microarrays and proteomics technologies. These advances have made it feasible to generate a truly comprehensive parts list for any organism and to track changes over time. Ultimately, it should be possible to enumerate all the informational units of the genomes (protein coding genes, non–protein coding genes, regulatory regions), their processed forms, and their dynamic presence in cells.

Rapid advances in large-scale data collection and analysis have given scientists a global yet detailed view of cellular processes, instead of focusing on individual molecules or a small number of interacting molecules. Unprecedented opportunities have emerged that may open the door to uncover hidden rules governing the ensemble of biomolecules working concertedly to perform certain functions in the cell. In the meantime, substantial challenges in information integration, interpretation, and representation have arisen. In order to move beyond the phase of cataloguing the parts list and truly transform data into knowledge, and knowledge into principles, iterative cycles of data collection and model generation and validation will be necessary.

A higher-order problem presents itself in understanding how the genome-encoded components and the other molecules (metabolites, ions, water, etc.) are constituted in networks of interacting molecules with particular distributions in time and space. Advances in imaging techniques and analytic methods are beginning to yield copious quantitative and spatial data on specific molecules in biological systems. Knowledge of the network and changes in its components over time, and the local rules by which the individual components distribute material and information, will substantially advance our knowledge.

At the organism level, phenotype must take into account the relationships and interactions of biological and environmental variables. Basic biological systems—including gene sequences, structures, and pathways that direct metabolism and development—vary within individuals, among individuals, among populations, and among species. Advances in complex systems-level understanding must ultimately include models that account for these variations.

Medical, biotechnological, and other uses of biological information increasingly depend on our ability to understand the principles and dynamics that explain the behavior of the system as a whole. Whether the goal is to understand the consequences of disease or injury, identify particular molecular targets for drug interventions, or modify the metabolism of microorganisms to produce medicines, the challenge is predictability. Predicting how the system of interest will respond to an intervention is a computational problem. For biological systems, this challenge is daunting.

Parallel to scientific challenges are organizational and educational challenges. At the institutional level, building cohesive multidisciplinary research teams by integrating expertise across traditional disciplinary boundaries is not a simple undertaking. Beyond institutions, excessive overlap and redundancy in project selection and tool development exists in the research communities that could be reduced by promoting communications, collaborations, and technology and data sharing. The emergence of new science demands an adequate workforce of new scientists. Training for the future leaders of systems biology research who are knowledgeable and skilled in both experimental and computational subjects is timely. Good mechanisms and plans to address these challenges are significant tasks of the centers.

High priority will be given to projects that integrate multi-investigator, multidisciplinary approaches with a high degree of interplay between computational and experimental approaches. Innovation is critical for both research project design and infrastructure design with a mission of serving communities beyond the participating investigators, institutions, and collaborators. A variety of organizational models are possible; it is not the intent of this RFA to prescribe any particular one.

The NIGMS awarded two centers under this program in 2002 (http://www.nigms.nih.gov/news/releases/complex_centers.html), two centers in 2003 (http://www.nigms.nih.gov/news/releases/complex_centers-2003.html), and one center in 2004 (http://www.nigms.nih.gov/news/releases/quantitative_bio_center.html). Potential applicants should become familiar with the research focuses of the existing centers. Research conducted by the future centers should complement and enhance projects already funded.

Some groups interested in the subject of this RFA might find the P01 mechanism more suited to the scale of their efforts; they should consult the prior announcement at http://grants.nih.gov/grants/guide/pa-files/PA-98-077.html.

The NIGMS intends to support systems biology research for the areas that are central to its mission of supporting basic biomedical research, and that focus on developing new computational approaches to biomedical complexity. Research areas that historically have been computationally based (e.g., molecular structure and modeling) are excluded as a focus of this center program. Research focusing on disease processes and their specific organ systems is not eligible. NIGMS mission areas include, but are not limited to, the following: 1) signaling networks and the regulatory dynamics of cellular processes such as cell cycle control, transient complex formation, organelle biogenesis, and intercellular communications; 2) supramolecular machines, such as the replisome, spliceosome, and molecular motor assemblies in cell division and motility; 3) pattern formation and developmental processes in model systems (e.g., *Drosophila*, *C. elegans*, etc.); 4) metabolic networks and the control of the flux of substrates, intermediates, and products in cell physiology; 5) organ system networks involved in multiorgan failure in shock, trauma, and burn injury; and 6) genetic architecture of biological complexity related to inherited variation and environmental fluctuations.

The NIGMS National Centers for Systems Biology will be expected to provide national leadership in systems biology research and training. To do so, they will be expected to support training and outreach activities that will ensure the flow of information and expertise both into and out of the centers. Centers should have plans to bring the most advanced technologies developed at other laboratories to the centers and to disseminate expertise and knowledge to a wider community through collaborations, visiting investigatorships, fellowships, center websites, workshops, symposia, summer courses/internships, and/or other means. To maximize the impact, centers should conduct training at multiple levels appropriate to their institutions. Incorporation of developmental research projects led by junior and new investigators into the center research and development plans is strongly encouraged. Over a period of time, centers should evolve into integrated research, training, and knowledge exchange headquarters of scientific communities that will be the engines for coordinated scientific discoveries. The centers should also have plans for outreach to undergraduate institutions, including minority-serving institutions. Information on relevant minority-serving institutions may be obtained by consultation with staff of the NIGMS Division of Minority Opportunities in Research (http://www.nigms.nih.gov/about_nigms/more.html).

In addition to research and training contributions, successful centers will provide their home institutions with the means to implement organizational and professional changes that will make systems biology research an attractive career option for both established and entry-level investigators.

This funding opportunity will use the NIH P50 Research Center Grant award mechanism. As an applicant, you will be solely responsible for planning, directing, and executing the proposed project. This RFA is a one-time solicitation and may be reannounced in the future. The earliest expected award date is in December 2005. Applications that were submitted in response to previous RFAs of this program but unfunded may be revised and resubmitted for this RFA.

The NIGMS intends to commit up to $7 million in fiscal year 2006 to fund one to three new P50 center grants in response to this RFA. An applicant may request a project period of up to five years and a budget for direct costs of up to $2 million per year, exclusive of subproject fiscal and administrative costs (see http://grants.nih.gov/grants/guide/notice-files/NOT-OD-04-040.html).

The PHS 398 application instructions are available at http://grants.nih.gov/grants/funding/phs398/phs398.html in an interactive format. For further assistance, contact GrantsInfo by calling 301-435-0714 or e-mailing GrantsInfo@nih.gov.

Applications must be prepared using the PHS 398 application instructions and forms (rev. 5/2001). Applications must have a Dun & Bradstreet (D&B) Data Universal Numbering System number as the universal identifier when applying for federal grants or cooperative agreements. This number can be obtained by calling 1-866-705-5711 or online at http://www.dnb.com/us/. The D&B number should be entered on line 11 of the face page of the PHS 398 form.

Letters of intent must be received by 25 January 2005, with 25 February 2005 the deadline for applications. The complete version of this announcement is available online at http://grants.nih.gov/grants/guide/rfa-files/RFA-GM-05-010.html#PartI.

Contact: James J. Anderson, Center for Bioinformatics and Computational Biology, NIGMS, 45 Center Dr, Rm 2As.25A, MSC 6200, Bethesda, MD 20892-6200 USA, 301-594-0943, fax: 301-480-2228, e-mail: andersoj@nigms.nih.gov; Jiayin (Jerry) Li, Center for Bioinformatics and Computational Biology, NIGMS, 45 Center Dr, Rm 2As.19F, MSC 6200, Bethesda, MD 20892-6200 USA, 301-594-0682, fax: 301-480-2004, e-mail: lij@nigms.nih.gov. Reference: RFA No. RFA-GM-03-009

## Environmental and Human Health Effects of Manufactured Nanomaterials

The purpose of this collaborative research program is to strengthen support by the Environmental Protection Agency (EPA), the National Science Foundation (NSF), and the National Institute for Occupational Safety and Health (NIOSH) of research on the potential implications of nanotechnology and manufactured nanomaterials on human health and the environment. Research areas of interest include the toxicology, fate, transport/transformation, and bioavailability of nanomaterials, as well as human exposures to these materials. Proposals should address one of these topics.

The EPA supports research to meet its mission of protecting the environment and human health. Information used in risk assessment, which comprises hazard identification and exposure assessment, is central to the EPA’s methods to meet its mission. As such, the EPA is interested in funding research on the possible risks and exposure routes of newly produced chemicals and materials at the nanoscale.

At the NSF, proposals should assist and enable the engineering and scientific communities to advance the frontiers of research, innovation, and education. The research should focus on emerging and potentially transformative research ideas, application of new expertise, or new approaches to established research topics.

NIOSH supports research to identify and investigate the relationships between hazardous working conditions and associated occupational diseases and injuries; to develop more sensitive means of evaluating hazards at work sites, as well as methods for measuring early markers of adverse health effects and injuries; to develop new protective equipment, engineering control technology, and work practices to reduce the risk of occupational hazards; and to evaluate the technical feasibility or application of a new or improved occupational safety and health procedure, method, technique, or system.

Nanotechnology has been defined by the interagency Subcommittee on Nanoscale Science, Engineering, and Technology of the federal Office of Science and Technology Policy as research and technology development at the atomic, molecular, or macromolecular levels, in the length scale of approximately 1- to 100-nanometer (nm) range, to provide a fundamental understanding of phenomena and materials at the nanoscale, and to create and use structures, devices, and systems that have novel properties and functions because of their small and/or intermediate size. The novel and differentiating properties and functions are developed at a critical length scale of matter typically under 100 nm. Nanotechnology research and development includes manipulation under control of the nanoscale structures and their integration into larger material components, systems, and architectures. Within these larger-scale assemblies, the control and construction of their structures and components remains at the nanometer scale. In some particular cases, the critical length scale for novel properties and phenomena may be under 1 nm (e.g., manipulation of atoms ~0.1 nm) or larger than 100 nm (e.g., nanoparticle-reinforced polymers have the unique feature at ~200–300 nm as a function of the local bridges or bonds between the nanoparticles and the polymer). See http://www.nano.gov/ for more information.

Many industries are currently involved in nanotechnology-related activities. Among these activities is the manufacture of nanoscale materials that are used in a wide range of products, such as sunscreens, composites, medical devices, and chemical catalysts. According to data collected by the National Nanotechnology Initiative, the quantity of manufactured nanoscale materials is expected to grow significantly in the next five years. Business Communications Company has projected a $10 billion global demand for nanoscale materials, tools, and devices in 2010. This large increase in demand and production could lead to environmental exposures of humans and other organisms to nanoscale materials.

There is a serious lack of information about the human health and environmental implications of manufactured nanomaterials, e.g., nanoparticles, nanotubes, nanowires, fullerene derivatives, and other nanoscale materials. Environmental and other safety concerns about nanotechnology have been raised. As part of the missions of the EPA and NIOSH to protect human health and the environment, this solicitation requests research proposals that address potential health and environmental concerns of nanomaterials using the best science available, in keeping with the missions of the NSF and the other agencies.

Potentially harmful effects of nanotechnology might arise as a result of the nature of the nanoparticles themselves, the characteristics of the products made from them, or aspects of the manufacturing process involved. The large surface area, crystalline structure, and reactivity of some nanoparticles may facilitate transport in the environment or lead to harm because of their interactions with cellular material. The size of nanomaterials could facilitate and exacerbate any harmful effects caused by the composition of the material.

Some research has been done on inhalational and dermal exposure to nanoparticles. However, the current research on ultrafine particles may not be applicable to manufactured nanoparticles because the ultrafine materials studied are neither a consistent size nor pure in chemical or structural composition. Exposure may occur via the dermal and ingestion routes, as well as the inhalational route. It is unknown whether nanomaterials bioaccumulate and thereby pose human health and environmental risks.

Little is known about the fate, transport, and transformation of nanomaterials after they enter the environment. As the production of manufactured nanomaterials increases and as products containing manufactured nanomaterials are disposed of, these materials could have harmful effects as they move through the environment.

The RFA sponsors are particularly interested in supporting research related to manufactured nanomaterials in the following areas:

- *Toxicology of manufactured nanomaterials.* What is the toxicity/potential toxicity of manufactured nanomaterials? Can similar nanomaterials be grouped with respect to their bioactivity? What are the health effects associated with nanomaterial mixed exposures or multiple exposure routes? What are the dose–response characteristics of nanomaterials? What are appropriate testing procedures, models, and biomarkers to evaluate the potential toxicological effects of nanomaterials in humans and/or other species in natural ecosystems? What extrapolation models are needed to evaluate or predict toxicity? What is the mode of action and mechanism of toxicity? What effects may occur in exposed human and wildlife populations? Are some subpopulations more sensitive to nanomaterials? Do nanoparticles impact ecological (animal/plant) receptors?
- *Environmental and biological fate, transport, and transformation of manufactured nanomaterials.* By what means do/can manufactured nanomaterials enter the environment? What are the modes of dispersion for nanomaterials in the environment? Do manufactured nanoparticles undergo transformation in the environment? Do manufactured nanoparticles bioaccumulate through the food chain?
- *Exposure and bioavailability of manufactured nanomaterials.* How and to what degree are humans exposed to nanomaterials in the environment and workplace? What effects may occur in exposed human populations and occupations? Are some subpopulations more vulnerable to nanomaterial exposure? What are the exposure pathways for humans? What are the effects of nanomaterials and mixtures on engineering controls and personal protective equipment? What releases might occur from the manufacturing processes of nanomaterials? At what stage in the product life cycle might exposure occur? How will changes from current processes to nanotechnology processes affect material flows of hazardous substances? What are the life cycle impacts from the manufacturing processes for nanomaterials?

Because the manufacturing of nanomaterials is not widespread and nomenclature is not standard, researchers must indicate in their proposals which nanomaterials they will use and where they will obtain them, including any needed collaboration with a materials manufacturing corporation or research lab that is synthesizing a commercially viable material. Thus, in the proposal, information on the source, potential use, composition, and present or future availability of the material being studied must be included. Researchers are encouraged to explore the appropriateness and availability of special nanotechnology user facilities at the Department of Energy; see http://www.nano.gov/html/centers/DOEcenters.html. The National Institute of Standards and Technology also offers user facilities; see http://www.nano.gov/html/centers/NISTcenters.html for information.

It is anticipated that a total of approximately $7 million will be awarded, depending on the availability of funds. The EPA intends to commit up to $5 million in fiscal year 2005, NIOSH intends to commit up to $1 million, and the NSF intends to commit up to $1 million. Depending on the proposal types, 16–20 awards may be given.

For a standard (e.g., R01 type) grant, an applicant may request a project period of up to three years and a budget for total costs (direct and indirect) not to exceed $400,000 total for a three-year period. For an exploratory (e.g., R21, SGER type) grant mechanism, an applicant may request a project period of up to two years and a budget for total costs (direct and indirect) not to exceed $200,000 total for a two-year period. Although the financial plans of the EPA, NIOSH, and the NSF provide support for this program, awards pursuant to this RFA are contingent upon the availability of funds and the receipt of a sufficient number of meritorious applications. Proposals with budgets exceeding the total award limits will not be considered. This RFA will use EPA, NIOSH, and NSF award mechanisms.

Applications must be received by 5 January 2005. The complete version of this announcement is available online at http://es.epa.gov/ncer/rfa/2004/2004_manufactured_nano.html.

Contact: Barbara Karn, EPA, 202-343-9704, e-mail: karn.barbara@epa.gov; Nora Savage, EPA, 202-343-9858, e-mail: savage.nora@epa.gov; Cynthia J. Ekstein, NSF, 703-292-7941, e-mail: cekstein@nsf.gov; Adele M. Childress, Office of Extramural Programs, NIOSH, CDC, 1600 Clifton Rd NE, Executive Park, Bldg 24, Rm 1427, MS E-74, Atlanta, GA 30333 USA, 404-498-2509, fax: 404-498-2571, e-mail: ahc0@cdc.gov. Reference: STAR-2005-B1

